# Is 25-Hydroxyvitamin D Associated with Glycosylated Hemoglobin in Patients with Type 2 Diabetes Mellitus in Saudi Arabia? A Population Based Study

**DOI:** 10.3390/ijerph18062805

**Published:** 2021-03-10

**Authors:** AlJohara M AlQuaiz, Abdullah A Alrasheed, Ambreen Kazi, Mohammad Ali Batais, Khaled M Alhabeeb, Amr Jamal, Mona A Fouda

**Affiliations:** 1 Reserach Chairs Program, Princess Nora Bent Abdullah Chair for Women’s Health Research, King Saud University, Riyadh 231831, Saudi Arabia; jalquaiz@ksu.edu.sa (A.M.A.); kmh-100@hotmail.com (K.M.A.); monafoudaneel@yahoo.com (M.A.F.); 2 Department of Family & Community Medicine, College of Medicine, King Saud University, Riyadh 7065, Saudi Arabia; aalrasheed1@ksu.edu.sa (A.A.A.); drmohammed34@gmail.com (M.A.B.); amrjamal@ksu.edu.sa (A.J.); 3 Endocrinology Division, Department of Medicine, College of Medicine, King Saud University, Riyadh 7065, Saudi Arabia

**Keywords:** HbA1c, 25-hydroxyvitamin D, Type 2 diabetes mellitus, Saudi Arabia

## Abstract

Background: Saudi Arabia has a high burden of diabetes mellitus and vitamin D deficiency. The objective of this study was to explore the association between glycosylated hemoglobin and 25-hydroxyvitamin D in patients with type 2 diabetes mellitus (T2DM) in Riyadh, Saudi Arabia. Methods: An interview based cross-sectional study was conducted on 606 patients with type 2 diabetes, aged 30–75 years, visiting primary health care centers. Blood samples were collected for measuring HbA1c, 25(OH)D and bone and lipid markers. Multivariable linear regression analysis was conducted to explore the association between HbA1c and 25(OH)D. Results: The mean (±SD) levels for HbA1c and 25(OH) D were 7.69 (±1.77) and 44.28 (±23.06), respectively. Around 55% of patients had uncontrolled HbA1c (>7.0), whereas vitamin D deficiency (<50 nmol/L) was found in 52.3% (=317). Multiple linear regression analysis found that a unit increase in vitamin D levels and parathyroid hormone levels was associated with −0.17 (−0.02, −0.01, *p* < 0.001) and −0.20 (−2.66, −1.18, *p* < 0.001) unit decrease in levels of HbA1c, respectively. Similarly, increasing age was associated with −0.15 (−0.01, −0.04, *p* = 0.002) unit decrease in HbA1c levels, whereas unit increases in serum alkaline phosphatase, calcium and diabetes duration were associated with 0.22 (0.01, 0.02, *p* < 0.001), 0.14 (1.03, 3.88, *p* = 0.001) and 0.26 (0.42, 0.78, *p* < 0.001) unit increase in HbA1c levels, respectively. Conclusion: HbA1c levels are associated with 25-hydroxyvitamin D levels. For better control of HbA1c levels, it is important to maintain 25-hydroxyvitamin D level and bone markers within normal range.

## 1. Introduction

Diabetes mellitus (DM) is a global epidemic, and it is estimated that, worldwide, 415 million people are suffering from DM [1,2]. A recent systematic review and meta-analysis from the Middle East has reported the prevalence of type 2 diabetes mellitus (T2DM) at about 14.6%, ranging from 2.6% to 21.9% [3]. Diabetes leads to drastic complications, accounting for significant morbidity, disability and mortality. Globally, over three million people die of diabetes and its complications every year [4,5]. Saudi Arabia ranks second among Middle Eastern countries in terms of DM, as it has seven million people with diabetes and three million with pre-diabetes, and it is estimated that the DM prevalence is on the rise [6]. 

Vitamin D deficiency has been reported to be associated with metabolic abnormalities, resulting in abnormal glucose metabolism, impaired insulin secretion and DM [7,8]. A meta-analysis of 28 studies, including 99,745 participants, that assessed the association between 25-hydroxyvitamin D (25(OH)D) levels and cardiometabolic disorders, has reported that high levels of vitamin D among middle-age and elderly populations reduced the risk of diabetes mellitus, metabolic syndrome and cardiovascular diseases (CVD) [9]. Another meta-analysis of 24 controlled trials, including 1528 individuals diagnosed with T2DM, has shown that optimum levels of serum 25(OH)D improve glycemic control and insulin resistance, hence, reducing HbA1c levels [10]. However, a systematic review of interventional studies revealed contradictory findings where the controlled trials showed positive impacts on glycemic control, insulin resistance and beta cell dysfunction, while longitudinal studies showed no such effects [11]. 

In Saudi Arabia, vitamin D deficiency is common, its prevalence ranging from 60% to 100% [10,11,12,13,14]. A study conducted at a tertiary care center in Riyadh reported an inverse relationship between HbA1c and vitamin D levels, in both patients with diabetes and pre-diabetes [15]. Conversely, Mousa et al. conducted a randomized placebo-controlled trial to investigate the effect of vitamin D supplementation on the improvement of insulin sensitivity or secretion in 65 overweight or obese patients. They reported no improvement with vitamin D supplementation in vitamin D deficient patients, rendering it an ineffective strategy to reduce diabetes risk [16]. The above studies reflect that there is insufficient and contradictory evidence of a beneficial role of vitamin D in patients with established T2DM. The objective of this study was to explore the association between glycosylated hemoglobin and 25-hydroxyvitamin D in patients with T2DM in Riyadh, Saudi Arabia.

## 2. Materials and Methods

### 2.1. Study Setting and Population

This study is part of WISHES project “Women in Saudi Arabia Health Examination Survey” a large cross-sectional survey conducted in Riyadh city, Saudi Arabia during 2015–2016. On average 18 primary health care centers (PHCCs) were randomly selected from each of the five administrative regions of Riyadh. All participants who had physician diagnosed T2DM, were Saudi nationals, residents of Riyadh and aged between 30 and 75 years were eligible to participate, whereas non-Saudis, people with mental illness and pregnant women were excluded from the study. Signed informed consent was taken from all participants before the interviews and collection of blood samples. The study protocol was approved by the Institutional Review Board, King Saud University (E-12–658) and the Institutional Review Board of the Ministry of Health, Dammam (IRB ID MOH0151). 

### 2.2. Data Collection Procedures

The interviews comprised detailed questions on sociodemographic characteristics (age, sex, education, occupation), smoking, medical history, diet intake, physical activity and family history of diabetes or vitamin D deficiency. Physical activity was measured using the translated and validated Arabic version of the International Physical Activity questionnaire (IPAQ-short form) [17]. 

### 2.3. Anthropometric and Blood Pressure Measurements

Anthropometric measurements included weight, which was measured using an electronic scale (Secca 220—Hamburg, Germany, 2009), and height, which was measured using a stadiometer. Body mass index (BMI) was calculated using the formula of weight in kg divided by height in meters square. Waist circumference (WC) was measured in cm at the mid-point between the lowest rib and top of the hip bone (iliac crest). Central obesity for men and women was defined as waist circumference of >90 and >80 cm, respectively [18]. Each participant’s blood pressure was measured twice with 5 to 10 min interval using the oscillometric method, according to the instruction manual (Omron-5 Series TM Blood Pressure Monitor Model BP742—China 2010). The average reading was taken for analysis.

### 2.4. Blood Collection Procedures

Fasting samples of venous blood were collected in three different vials: two with yellow caps and one purple cap. In total, 5 cc of venous blood was collected in the first yellow cap for measuring cholesterol, lipids and triglycerides (TG), alkaline phosphatase (ALP), calcium (Ca), phosphorus and albumin; 5 cc in a 2nd yellow cap for measuring 25(OH)D and parathyroid hormone (PTH) and 3 cc in purple cap test tube with EDTA for measuring HbA1C. Test tubes were placed in a labelled plastic bag and then refrigerated at a temperature of −2 to 8 degrees Celsius. The samples were transported to King Khalid University hospital laboratory, by using cold storage boxes.

#### 2.4.1. Measurement of Standardized Vitamin D 25(OH)D, Parathyroid, Calcium and Alkaline Phosphatase Levels

Measurement of 25(OH)D was conducted in two steps. Initially, direct serum 25(OH)D levels were measured using electrochemiluminiscence (ECLIA immunoassay, Modular Analytics E170, Roche Diagnostics GmbH, Mannheim, Germany). The intra assay and inter assay coefficients of variation (CV) for serum 25(OH)D were 6.8% and 13.1%, respectively. This was followed by standardization of serum 25(OH)D, using the automated Roche Elecsys Cobas e411 analyzer (Roche Diagnostics, GmbH, Mannheim, Germany) by means of electrochemiluminescence immunoassay [19]. Parathyroid (PTH) levels were measured using an electro-chemiluminescence assay (ECLIA immunoassay, Modular Analytics E170, Roche Diagnostics GmbH, Mannheim, Germany) with a hospital standard laboratory reference range of 1.6–6.9 pmol/L. The intra assay and inter assay CVs were 2.0% and 3.4%, respectively. Serum calcium (corrected for albumin binding) was measured in mmol/L and serum alkaline phosphatase (ALP) was measured in units per liter (U/L) using routine chemical analyzers (Siemens Stream Lab RxL Max, Erlangen, Germany).

#### 2.4.2. Measurement of Glycated Hemoglobin

HbA1c was measured using ion exchange high-performance liquid chromatography method (HPLC) using the Tosoh G8 HPLC Analyzer (Manufacturer Tosoh Bioscience, Inc, South San Francisco, CA, USA). The coefficient of variation was less than 2%. According to the American Diabetic Association, the diagnostic cut off point for defining uncontrolled HbA1c in patients with diabetes is taken as ≥7.0 [20]. 

#### 2.4.3. Measurements of Lipid Level

Serum levels for total cholesterol, high density lipoproteins (HDL) and triglycerides (TG) were measured in mmol/L by a fully automated analyzer (Siemens Dimension RxL, Marburg, Germany) using enzymatic methods. Low density lipoprotein (LDL) was calculated in mmol/L by utilizing the formula (Total cholesterol-HDL-TG/5)) [21]. The intra assay and inter assay coefficients of variation for cholesterol, HDL and TG were 0.84 and 1.30, 1.9 and 2.1, and 0.4 and 1.0, respectively. 

### 2.5. Ethical Considerations

The study protocol was approved by the Institutional Review Board, King Saud University (E-12–658) and the Institutional Review Board of the Ministry of Health, Saudi Arabia (IRB ID MOH0151). Written informed consent was obtained from participants after explaining the purpose of the study, with emphasis on voluntary participation, anonymity and confidentiality.

### 2.6. Statistical Analysis

The data were analyzed using Statistical Package for Social Sciences (SPSS^®^ version 21, IBM Corp., Armonk, NY, USA). Descriptive statistics include proportions for categorical and mean (±standard deviation) for continuous variables. Mean values were replaced by median (interquartile range) values for variables with skewed distribution. HbA1c >7.0% was defined as an uncontrolled level, whereas vitamin D < 50 nmol/L was defined as deficiency. Log transformation was conducted for skewed variables before entering them in the regression analysis. Univariate and multivariate linear regression analysis calculated the unadjusted and adjusted beta coefficients and the 95% confidence interval for the association between HbA1c with vitamin D, and bone and lipid markers. The association was adjusted for sex, BMI and cholesterol and TG. The level of significance was taken at *p* < 0.05. 

## 3. Results

The study sample comprised 606 Saudi participants; 164 (27%) men and 442 (73%) women) with physician diagnosed T2DM. All patients were adults, with mean age of 53.98 (±10.27) years, ranging from 30 to 70 years. The majority (80%) were currently married, and around 50% had a low level of education. Women were mostly housewives, whereas 10% of men were retired. Only 5% were current smokers, whereas around 70% reported low level physical activity. 

Table 1 shows the mean (±SD) for age, anthropometric measurements, and blood markers. The mean BMI in the study population was 32.34 (±6.11), with 66% obese and 25% overweight (BMI ≥30 and ≥25 kg/m^2^, respectively). The mean BMI for women was higher in comparison to men (33.0(±5.8) vs. 30.5(±6.6) kg/m^2^ (*p* < 0.001)). The majority (85.5%) of patients were suffering from central obesity, with no significant difference between men and women (defined as waist circumference in men >90 and women >80 cm). The mean systolic and diastolic blood pressure were on the higher side, i.e., 130.38 (±17.85) and 76.55 (±11.51) mmHg, respectively. 

Based on the international cut-off for controlled HbA1c at 7%, we found that around 55% (*n* = 331) of patients had HbA1c >7.0%. The mean age of patients with diabetes was 8.8 (±7.0) years. Around 74% (*n* = 443) of patients mentioned positive family history for diabetes. The mean (±SD) for 25(OH)D was 44.28 (±23.06). Around 66% (*n* = 398) of the sample were suffering from low vitamin D (<50 nmol/L), and 19% (*n* = 114) had a positive history of osteoporosis. Increased mean values for 25(OH) D were prevalent among young adults, as compared to the elderly (>60 years) (vitamin D: 37.3 ± 21.3 vs. 50.1 ± 23.8 nmol/L, (*p* < 0.001). Around 21% of patients were taking vitamin D supplements regularly. Around 16% (*n* = 98) had PTH values >6.9 pmol/L, whereas 11.6% (*n* = 70) and 7.4% (*n* = 45) had raised alkaline phosphatase and phosphorus, respectively. Almost all (99.7%) diabetic patients had normal calcium values, except for two patients with low calcium levels. Almost half (48%, *n* = 294) of the diabetic patients had raised triglyceride levels (>1.48 mmol/L). Similarly, 39% (*n* = 239) had elevated cholesterol levels (>5.2 mmol/L). 

Table 2 shows the univariable linear regression analysis revealing unit decreases of −0.12 (−0.02, −0.003, *p* = 0.003) and −0.15 (−2.15, −0.68, *p* < 0.001) in HbA1c (dependent variable) for unit increases in vitamin 25(OH)D and parathyroid hormone, respectively. Conversely, both calcium and alkaline phosphatase were positively associated with HbA1c, causing 0.15 (1.35, 4.15, *p* < 0.001) and 0.22 (0.01, 0.02, *p* < 0.001) unit increases in HbA1c, respectively. Similarly, lipid markers found a significant positive association between unit changes in cholesterol and triglycerides causing 0.09 (0.02, 0.28, *p* = 0.03) and 0.21 (1.01, 2.20, *p* < 0.001) unit increases in HbA1c levels. Age, sex, BMI and intake of sugary items were not significantly associated with HbA1c.

**Figure 1:**
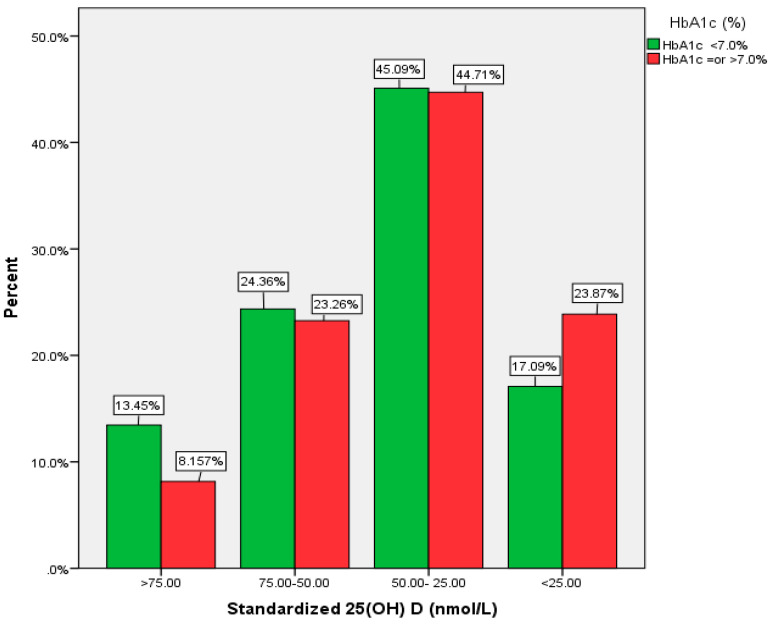
Proportion of diabetic patients with HbA1c < 7.0 and > 7.0% by vitamin D categories in Riyadh, Saudi Arabia.

The association between HbA1c and vitamin D is supported by categorical data. Figure 1 shows the percentage of diabetic patients with uncontrolled HbA1c, by vitamin D categories. In diabetic patients with 25(OH)D <25.0 nmol/L, the proportion of those with HbA1c ≥7% was higher compared to HbA1c <7.0 (23.87% vs. 17.09). Similarly, in diabetic patients with 25(OH)D >75.0 nmol/L, the proportion of those with HbA1c <7.0 was higher compared to HbA1c ≥7% (13.45% vs. 8.16%). However, the *p* value was not significant (*p* = 0.06).

Table 3 shows the multivariate linear regression analysis model for the association between 25(OH)D and HbA1c. We found that unit increases in vitamin D levels and parathyroid hormone levels were associated with −0.17 (−0.02, −0.01, *p* < 0.001) and −0.20 (−2.66, −1.18, *p* < 0.001) unit decreases in levels of HbA1c. Similarly, increasing age was associated with a −0.15 (−0.01, −0.04, *p* = 0.002) unit decrease in HbA1c levels, whereas unit increases in serum alkaline phosphatase, calcium and duration with diabetes were associated with 0.22 (0.01, 0.02, *p* < 0.001), 0.14 (1.03, 3.88, *p* = 0.001) and 0.26 (0.42, 0.78, *p* < 0.001) unit increases in the Hba1c levels, respectively. The model was adjusted for confounders, like sex, cholesterol and triglycerides. The R square value was 0.21.

## 4. Discussion

The significant correlation between HbA1c and bone and lipid markers is in support of past studies [22,23]. Previous studies have found that the active form of vitamin D, 25(OH)D, facilitates glucose transport into muscle cells, regulates insulin receptor gene expression and suppresses the expression of the rennin gene [24,25]. Moreover, detection of vitamin D receptors (VDR) on β cells has supported the role of 25(OH)D in the pathogenesis of diabetes [26]. These receptors are also found in skeletal and adipose tissues, which are involved in glucose homeostasis. Hence, it is evident that vitamin D affects glucose homeostasis through reduced insulin secretion, increased insulin resistance and hyperglycemia [26]. Studies show contradictory findings for the association between HbA1c and vitamin D, maybe because of differences in the levels of vitamin D. A significant increase in the number of patients with HbA1c ≥7.0% was observed only in the group with vitamin 25(OH)D <25 nmol/L, and not for those having vitamin D between 75.0–25.0 nmol/L [11]. 

The 25(OH)D levels are not only associated with direct glycemic control, as studies have revealed that vitamin D deficiency is a risk for severe complications of diabetes such as retinopathy among patients with well-controlled diabetes [27]. However, the bidirectional relationship between vitamin D and HbA1c cannot be ruled out. This notion was supported by one study when 25(OH)D levels were improved after hyperglycemia treatment [28]. In consensus with our results, several experimental and interventional studies have demonstrated that vitamin D supplementation can improve HbA1c and insulin resistance in patients suffering from T2DM [29]. We observed that patients taking vitamin D supplements had a lower mean of HbA1c (but not statistically significant) in comparison to those without supplements (7.49 ± 1.7 vs. 7.74 ± 1.9, *p* = 0.16).

It is important to note that the association between HbA1c and 25(OH)D is affected by bone markers, like PTH and calcium [30,31]. Normal physiological processes explain that decreased vitamin D leads to an increase in PTH levels, which normalizes the low calcium level [32]. This low calcium level is found to improve glucose tolerance and improve insulin sensitivity and secretion. However, a study from the USA found that increased PTH is associated with increased incidence of T2DM in white people but no association was observed in Black people [33], hence explaining the differences in results based on race and ethnicity. This may explain our findings, where increased PTH was associated with better control of HbA1c. However, future research is required to study the effect of ethnicity on bone and its association with T2DM, especially in the Arab region. Literature is available on the role of bone specific alkaline phosphatase in relation to glucose metabolism [34]. It is proposed that alkaline phosphatase may be the link between insulin resistance and vascular calcification, cardiovascular diseases or even mortality [34]. 

The association with lipids was significant on univariate analysis. A review study mentioned that 75% of patients with T2DM suffer from dyslipidemia [35], increasing the risk of developing cardiovascular diseases. Insulin controls the lipid level in blood by inhibiting lipolysis of stored fat in the adipose tissue. This decreases gluconeogenesis in the liver and stimulates the shifting of extracellular glucose into muscle cells for various metabolic activities [36,37]. Similar to our results, an in vivo study concluded that hypertriglyceridemia has no association with hyperglycemia, but the study findings are limited due to absence of control group and small sample size [38]. 

It is a common belief that diabetes is a disease of the elderly population. However, the inverse association with age suggests that HbA1c control is better among the elderly [39]. Hence, we suggest that awareness, self-care and screening should be initiated for all ages [39,40].

Although several educational campaigns targeting vitamin D deficiency are being conducted [41,42], the majority of patients had 25(OH)D <50 nmols/L, highlighting the need for health awareness. It is important for clinicians to inform their patients with diabetes about the association between HbA1c and 25(OH)D, and more so to maintain the 25(OH)D levels above the criterion value of 50 nnmol/L.

### Strengths and Limitations

The major strength of this study was that it included all socioeconomic, ethnic, working and non-working groups. Apart from daytime data collection that coincided with office timings, due to which a lower number of men participated in the study, we did not encounter any major difficulties in conducting this survey. Certain methodological issues do limit the generalizability of our findings. This study was cross-sectional in design; hence, it is difficult to establish a temporal relationship between HbA1c and 25(OH)D. Hemoglobin levels affect HbA1c levels; however, we did not measure hemoglobin levels, and hence, it could not be adjusted for. In addition, there may be some residual confounders that were not adjusted for in the analysis.

## 5. Conclusions

HbA1c levels are associated with bone health. Interventional studies with required intake of vitamin D supplements and specific diet can help us in understanding the role of nutrients in controlling HbA1c. In addition, educating patients on healthy lifestyle can help in controlling dyslipidemia, hence decreasing the risk for cardiovascular diseases. 

## Figures and Tables

**Table 1:** Mean (standard deviation) for age, anthropometric and blood markers for patients with diabetes in Riyadh, Saudi Arabia.

Variables	Mean (±SD)
Age (in years)	53.98 (±10.27)
Body Mass Index	32.34 (±6.11)
Waist circumference (cm)	99.33 (±13.31)
Blood Pressure (mmHg)	
Systolic	130.38 (±17.85)
Diastolic	76.55 (±11.51)
**Blood markers**	
HBA1C (%)	7.69 (±1.77)
Standardized 25 (OH) D (mmol/L)	44.28 (±23.06)
Parathyroid hormone (pmol/L) Median & IQR *	4.69 (IQR 2.5)
Corrected Calcium (mmol/L)	2.34 (±0.09)
Alkaline phosphatase (U/L)	104.15 (±29.40)
Phosphorus (mmol/L) Median & IQR	1.20 (IQR 0.23)
Total cholesterol (mmol/L)	4.93 (±1.04)
LDL-C (mmol/L)	2.89 (±0.97)
HDL-C (mmol/L)	1.23 (±0.33)
Triglycerides (mmol/L) Median and IQR	1.45 (IQR 1.06)

* IQR = Interquartile range.

**Table 2:** Univariable linear regression analysis showing unadjusted beta and 95% confidence interval (CI) for variables associated with HbA1c in Saudi patients with diabetes in Riyadh, Saudi Arabia.

Variable	B	Unadjusted Beta	95% Confidence Interval	*p* Value
**25-Hydroxyvitamin D (nmol/L)**	**−0.01**	**−0.12**	**−0.02, −0.003**	**0.003**
**Age (in years)**	0.01	0.08	−0.03, 0.001	0.06
**Sex**	0.05	0.01	−0.27, 0.37	0.76
**Body mass Index (kg/m^2^)**	−0.03	−0.06	−0.04, 0.01	0.12
**Physical Activity**	−0.28	−0.07	−0.58, 0.02	0.07
**Sugary items**	0.14	−0.07	−5.07, 0.22	0.07
**Duration with Diabetes (in years)**	**0.52**	**0.23**	**0.35, 0.70**	**<0.001**
**Parathyroid hormone (pmol/L) (log transformed)**	**−1.41**	**−0.15**	**−2.15, −0.68**	**<0.001**
**Corrected Calcium (mmol/L)**	**2.75**	**0.15**	**1.35, 4.15**	**<0.001**
**Alkaline phosphatase(µ/L)**	**0.01**	**0.22**	**0.01, 0.02**	**<0.001**
**Total Cholesterol** **(mmol/L)**	**0.15**	**0.09**	**0.02, 0.28**	**0.03**
**HDL–Cholesterol (mmol/L)**	−0.40	−0.07	−0.82, 0.02	0.06
**Triglycerides (mmol/L) (log transformed)**	**1.60**	**0.21**	**1.01, 2.20**	**<0.001**

The values in bold show the significant results.

**Table 3:** Multivariable linear regression model showing adjusted odds ratio and 95% confidence interval (CI) for the association between HbA1c and standardized 25-hydroxyvitamin in patients with diabetes in Riyadh, Saudi Arabia.

Variable	B	Adjusted Beta	95% CI	*p* Value
**Standardized 25(OH)D** **nmol/L**	**−0.01**	**−0.17**	**−0.02, −0.01**	**<0.001**
**Parathyroid hormone** **pmol/L**	**−1.92**	**−0.20**	**−2.66, −1.18**	**<0.001**
**Calcium (mmol/L)**	**2.45**	**0.14**	**1.03, 3.88**	**0.001**
**Alkaline phosphatase** **u/L**	**0.01**	**0.22**	**0.01, 0.02**	**<0.001**
**Age in years**	**−0.03**	**−0.15**	**−0.04, −0.01**	**<0.001**
**Duration with Diabetes (in years)**	**0.60**	**0.26**	**0.42, 0.78**	**<0.001**

Model adjusted for sex, cholesterol, triglycerides. The values in bold show the significant results.

## Data Availability

Data can be made available on special request to the Principal Investigator.
